# Mitochondrial Dynamics Associated with Oxygen-Glucose Deprivation in Rat Primary Neuronal Cultures

**DOI:** 10.1371/journal.pone.0063206

**Published:** 2013-05-02

**Authors:** Edina A. Wappler, Adam Institoris, Somhrita Dutta, Prasad V. G. Katakam, David W. Busija

**Affiliations:** 1 Department of Pharmacology, Tulane University School of Medicine, New Orleans, Louisiana, United States of America; 2 Department of Physiology, School of Medicine, University of Szeged, Szeged, Hungary; University of Iowa, United States of America

## Abstract

Our objective was to investigate the mitochondrial dynamics following oxygen-glucose deprivation (OGD) in cultured rat cortical neurons. We documented changes in morphology, protein expression, and DNA levels in mitochondria following OGD and examined the roles of mitochondrial fission [dynamin-related protein 1 (Drp1), fission protein-1 (Fis1)] and fusion [mitofusin-1 (Mfn1), mitofusin-2 (Mfn2), and optic atrophy-1 protein (OPA1)] proteins on mitochondrial biogenesis and morphogenesis. We tested the effects of two Drp1 blockers [15-deoxy-Δ12,14-Prostaglandin J_2_ (PGJ_2_) and Mitochondrial Division Inhibitor (Mdivi-1)] on mitochondrial dynamics and cell survival. One hour of OGD had minimal effects on neuronal viability but mitochondria appeared condensed. Three hours of OGD caused a 60% decrease in neuronal viability accompanied by a transition from primarily normal/tubular and lesser number of rounded mitochondria during normoxia to either poorly labeled or small and large rounded mitochondria. The percentage of rounded mitochondria remained the same. The mitochondrial voltage-dependent anion channel, Complex V, and mitoDNA levels increased after OGD associated with a dramatic reduction in Drp1 expression, less reduction in Mfn2 expression, an increase in Mfn1 expression, with no changes in either OPA1 or Fis1. Although PGJ_2_ increased polymerization of Drp1, it did not reduce cell death or alter mitochondrial morphology following OGD and Mdivi-1 did not protect neurons against OGD. In summary, mitochondrial biogenesis and maintained fusion occurred in neurons along with mitochondrial fission following OGD; thus Mfn1 but not Drp1 may be a major regulator of these processes.

## Introduction

Mitochondria undergo fission and fusion under physiologic conditions to maintain optimal morphological characteristics necessary to match ATP production to cellular needs. Maintaining a balance between fission and fusion is important in neurons because of high neuronal energy demand and long mitochondrial transport distances especially in motor neurons [1], [2]. Therefore, in neural cells the balance shifts toward fission compared with non-neural cells in order to maintain small, highly motile mitochondria consistent with need [2]. We postulated that unique neuronal requirements necessitate a different mode of mitochondrial dynamics regulation compared with other cell types especially under stress conditions. The major proteins involved in fission/fusion are dynamin-related protein 1 (Drp1), mitofusin-1(Mfn1), mitofusin-2 (Mfn2), and optic atrophy-1 protein (OPA1). Dynamin-related protein 1 induces mitochondrial fission after translocating to the mitochondrial outer membrane and polymerizing and binding with fission protein 1 (Fis1) [3], [4] with Drp1 activity regulated by post translational modificationssuch as phosphorylation [3], [5], [6].

Previous studies have shown that mitochondrial fragmentation, in some cases due to increased activity of fission proteins, is involved in apoptotic cell death pathology [4], [7]–[10], intensifying programmed cell death. Although mitochondrial fragmentation reduces ATP production, enlarged mitochondria due to an imbalance favoring fusion over fission, produce more energy compared with normal mitochondria [11], [12]. However, the opposite has also been reported [13], [14].

Under control conditions the Drp1 protein is present largely unassembled in the cytosol [15]. However, stress is known to cause assembly, oligomerization of Drp1 and transfer onto the mitochondria, where it induces membrane constriction and fission in most cell types [15], [16]. Recent evidence also showed that blocking Drp1 fission protein using mitochondrial division inhibitor-1 (Mdivi-1) can be protective against ischemia/hypoxia [16]–[19]. However, the effect of 15-deoxy-D12,14-prostaglandin J_2_ (PGJ_2_), which inhibits the GTPase activity of Drp1, on cell survival following stress is debated [20], [21].

Our study investigated mitochondrial dynamics in rat primary cortical neurons exposed to oxygen-glucose deprivation (OGD) and examined whether blocking mitochondrial fission influences cell survival following hypoxic insult. We investigated the effect of 3 h OGD on mitochondrial biogenesis from 0 h to 24 h following reoxygenation in neurons to determine: (1) mitochondrial fission (Drp1 and Fis1) and fusion (Mfn1, Mfn2, and OPA1) protein changes with western blot (WB); (2) changes in mitochondrial protein expression measuring respiratory chain complex proteins (II, V) and the voltage-dependent anion channel (VDAC) protein using WB; (3) changes in mitochondrial number by measuring the cellular level of mitochondrial DNA (mtDNA) copies using real-time PCR (rtPCR); (4) mitochondrial morphology using laser confocal microscopy (live mitochondrial staining) and transmission electron microscopy (TEM); and (5) mitochondrial dynamics after pharmacological blockage of Drp1 with PGJ_2_, and Mdivi-1. In addition, we investigated the effect of 1 h OGD on mitochondrial dynamics because of its modest effect on neuronal viability.

## Materials and Methods

### I. Primary Rat Cortical Neuronal Culture

Timed pregnant Sprague–Dawley rats were purchased from Harlan (Indianapolis, IN, USA). Animal use was approved by Wake Forest University Health Sciences Animal Care and Use Committee and by Tulane University Animal Care and Use Committee (IACUC protocol number: 4213). Under deep anaesthesia using 4–5% isoflurane (Baxter, Deerfield, IL, USA) in 70% N_2_O_2_/30% O_2_, the dam and E18 rat fetuses were euthanized and decapitated. Fetal brains were used for primary rat cortical neuron isolation and cultured as previously described [22], [23]. Following brain trituration, isolated neurons were plated onto poly-d-lysine coated 96-well plates (for viability) or dishes (for Western Blot, real time PCR, and EM) at a density of 10^6^ cells/cm^2^ or onto poly-l-lysine coated glass coverslips for confocal analysis at a density of 2×10^5^ cells/cm^2^. Poly D-lysine coated plastics and coverslips were purchased from BS Biocoat (Franklin Lakes, NJ, USA); other cell culture plastics were purchased from Becton–Dickinson (San Jose, CA, USA), Santa Cruz Biotechnology (Santa Cruz, CA, USA), or TPP Techno Plastic Products AG (Trasadingen, Switzerland). The plating medium consisted of 60% Dulbecco’s modified Eagle medium (DMEM; Gibco BRL, Grand Island, NY, USA), 20% Ham’s F-12 Nutrient Mixture (Gibco), 20% horse serum (Gibco), l-glutamine (0.5 mM; SIGMA, St. Louis, MO, USA), and Penicillin-Streptomycin (1 µL/mL; SIGMA). Cultures were maintained in a humidified 5% CO_2_ incubator. After cell attachment, plating medium was replaced with feeding medium: Neurobasal medium (Gibco), B27 supplement (2%; Gibco), l-glutamine (0.5 mM, SIGMA), 2-mercaptoethanol (55 µM; Gibco), and KCl (25 mM: SIGMA). Cytosine β-D-arabinofuranoside (ARA-C, SIGMA) was added to the feeding medium for 3 days in 1 µM concentration. Microtubule-associated protein-2 (MAP-2) and glial fibrillary acidic protein (GFAP) immunostaining certified the purity of the cultured neuron on the 7^th^ day *in vitro*. For primary antibody information see Table 1, for secondary antibody information see western blot and immunostaining protocols.

**Table 1:** Antibodies used in this study.

Against	Type	Firm	Application	Dilution
Drp1	Mouse	BD Transduction Laboratories (Clone 8, #611112)	WB	WB 1∶4000
Phospho-Drp1 (Ser616)	Rabbit	Cell Signaling (#3455)	WB	WB: 1∶1000
Drp1	Rabbit	Novus Biologicals (#NB110-55237)	WB	WB: 1∶1000
Fis1	Rabbit	ENZO Life Sciences (#ALX 210-907-R100)	WB	WB: 1∶500
Mfn1	Mouse	Novus Biologicals (#NBP1-71775)	WB	WB 1∶1000
Mfn2	Rabbit	SIGMA (M6444)	WB	WB: 1∶2000
OPA1	Mouse	BD Transduction Laboratories (Clone 18, #612606)	WB	WB: 1∶4000
Complex II 70 kDa	Mouse	Invitrogen (#459200)	WB	WB: 1∶10000
Complex V Subunit Alpha	Mouse	Invitrogen (#459240) 55 kDa	WB	WB: 1∶2000
MAP2	Mouse	BD (610460)	IH	IH: 1∶500
VDAC	Rabbit	Cell Signaling (#4866)	WB	WB: 1∶500
GFAP	Mouse	BD (610565)	IH	IH:1∶500
β-Actin	Mouse	SIGMA (A5441)	WB	WB 1∶5000

### II. Oxygen Glucose Deprivation (OGD)

Oxygen glucose deprivation was induced nine days after neuron isolation and culturing. All culture plates/dishes/coverslips were washed with 1xphosphate buffered saline (PBS; SIGMA) and the culture medium was replaced with glucose-free Earle’s balanced salt solution (EBSS). Cultured neurons were placed in a ShelLab Bactron Anaerobic Chamber (Sheldon Manufacturing, Cornelius, OR) filled with Anaerobic Mixed Gas (AMG: 5% CO_2_-5% H_2_-90% N_2_) at 37°C for 1 or 3 h. The 5% H_2_ in the AMG removed the remaining traces of oxygen-forming water on a platinum catalyst. Oxygen levels were continuously monitored with an infrared gas analyzer (model 3750; Illinois Instruments, Ingleside, IL) and maintained below 1% O_2_. Control cell cultures were incubated in glucose-containing (1 mg/mL) EBSS in a regular 5% CO_2_ cell culture incubator. The OGD ended when cells were removed from the anoxic chamber and EBSS was replaced with regular feeding medium. Cells were washed twice with PBS before being returned to feeding medium and were replaced to a regular 5% CO_2_ incubator. Cells were washed with PBS and processed without further culture medium replacement for immediate sample collection.

### III. Treatment Protocols

Increasing doses of PGJ_2_ (Cayman Chemical, Ann Arbor, MI, USA) or Mdivi-1 (from ENZO Life Sciences, Farmingdale, NY, USA, and from Sigma) or vehicle were administered during the 3 h OGD or its 3 h control in EBSS or EBSS+glucose, respectively. The PGJ_2_ concentrations were 2.5–20 µM, whereas Mdivi-1 concentrations were 10–100 µM. For protease inhibition we used a protease inhibitor cocktail (Sigma, Cat# P1860) in a 5 µL/mL final concentration added to the EBSS solution during OGD.

### IV. Quantification of Cellular Viability

Cell viability was measured 24 h after OGD or control treatment using the tetrazolium-based CellTiter 96 AQ_ueous_ One Solution assay (Promega, Madison, WI, USA). This is a colorimetric assay based on the evidence that living cells containing NADH or NADPH can convert 3- (4,5-dimethylthiazol-2-yl) -5- (3-carboxymethoxyphenyl) -2- (4-sulfophenyl) -2H- tetrazolium inner salt to a formazan product. The undiluted, warm solution (20 µl) was added to the culture medium and incubated for 1 h at 37°C followed by measurement of absorbance at λ_abs_ = 492 nm with a FLUOstar OPTIMA microplate reader (BMG Labtech, Offenburg, Germany). Results were compared with sister cultures exposed to the same drug treatments on the same day and every cell culture plate contained untreated wells. Cell viability was calculated as the percentage of the untreated corresponding control culture wells (non-OGD) using the following formula: %viability_SAMPLE_ = (absorbance_SAMPLE_−absorbance_BACKGROUND_) ×100/(absorbance_CONTROL_−absorbance_BACKGROUND_). Background was measured in cell free, culture medium containing wells.

### V. Western Blot Analysis

Proteins were harvested by scraping neurons in ice-cold NP40 lysis buffer (Invitrogen) supplemented with proteinase inhibitor cocktail (Sigma, Cat# P8340) and phosphatase inhibitor cocktail (Sigma, Cat# P2850) (each 5 µl/mL). Cell lysates were resolved using two protocols: *standard protocol* (denaturing conditions) and *non-denaturing protocol* (non-denaturing conditions). The standard protocol was used unless stated otherwise; the non-denaturing protocol was used for protein oligomerization/degradation investigations. For the standard protocol, cells were sonicated and equal amounts of protein (15 µg) from the whole cell lysates were incubated with sodium dodecyl sulfate (SDS)/β-mercaptoethanol sample buffer at 100°C for 5 min. For the non-denaturing protocol, cells were homogenized on ice in a glass homogenizer and frozen-thawed twice using liquid nitrogen followed by incubating equal amounts of protein (15 µg) in a β-mercaptoethanol free, lithium dodecyl sulfate (LiDS) sample buffer at room temperature for 5 min. Protein samples were separated by electrophoresis on a 4–20% SDS-PAGE gradient gel and proteins were transferred onto a PVDF (0.22 µm) or nitrocellulose membrane. Membranes were then incubated in a blocking buffer (Tris-buffered saline, 0.1% Tween 20 and 1% skimmed milk powder) for 1 h at room temperature followed by incubation with primary antibodies overnight at 4°C in the blocking solution. The membranes were washed three times in Tris-buffered saline with 0.1% Tween 20 and incubated for 1 h in the blocking buffer with goat anti-rabbit IgG (1∶5000, Santa Cruz) or goat anti-mouse IgG (1∶5000, Santa Cruz) conjugated to horseradish peroxidase. The final reaction products were visualized using enhanced chemiluminescence (SuperSignal West Pico; Pierce, Rockford, IL, USA) and developed on an X-ray film. For quantitative analysis, the bands were scanned and band densities were quantified using Image J 1.3.1 software. The band intensities were normalized to that of β-actin and the examined protein’s normalized level in the untreated control group was considered 100%.

### VI. Imaging

#### VI. a. Mitochondrial staining studies using confocal microscopy

We used the Chromeo™ Live Cell Mitochondrial Staining Kit for live mitochondrial staining (Active Motif, Carlsbad, CA, USA), which contains a water-soluble, non-toxic mitochondrial dye. Neurons were incubated with the dye (1∶5000) dissolved in feeding medium for 1 h in the cell culture incubator followed by two washes with PBS, cells then received feeding media, and were transferred later (2 h or 23 h) to a glass chamber slide containing phenol-free DMEM for live imaging. The coverslips were kept in 3.5 cm dishes and transferred to chamber-slides without re-plating. A Zeiss confocal microscope (Germany) was adjusted for the dye’s wavelengths (excitation: 554, emission: 568) and imaging parameters were unchanged during the same experiment.

To analyze mitochondrial network with live mitochondrial staining in neurons we investigated 50–100 cells/experiment; at least three independent experiments were averaged using untreated normoxic controls for each series. For the mitochondrial network analysis we defined four cell types: (1) normal, tubular/short, tubular; (2) rounded; (3) highly interconnected/long, tubular; and (4) poorly labeled. Please note that our cultured neurons do not have long, tubular mitochondria under normal conditions. This is in line with previous studies [2]. Cell category ratios were calculated for each experimental condition. To prevent bias the analysis was performed by a blinded investigator.

#### VI.b. Ultrastructural studies with transmission electron microscopy (TEM)

Cells were fixed in 2.5% glutaraldehyde (Sigma) in 0.1 M PBS for 30 min, and then washed three times with PBS. They were post-fixed in 1% osmium tetroxide in 0.1 mol/L phosphate buffer (pH 7.4), dehydrated in graded ethanol series, centrifuged at 14000 rpm for 2 min, and the pallet was embedded in Epon812. Ultrathin sections (80–90 nm) were mounted on formvar-coated nickel grids (200 mesh), air dried, and stained with 4.7% uranyl acetate and lead citrate (10 min and 2 min, respectively). The sections were put on grids and investigated using a FEI Tecnai BioTwin 120 keV TEM with a digital imaging setup (Wake Forest University).

### VII. RT-PCR

#### VII.a. mtDNA quantification

The DNA were harvested by scraping in ice-cold Nuclei Lysis Solution (200 µl/dish) from the DNA purification kit (Wizard SV Genomic DNA Purification System, Promega). For DNA extraction we followed the manufacturer’s instructions for the kit. The levels of mtDNA were measured by normalizing the mitochondrial cytochrome b gene (MT-CYB, Rn03296746_s1) to the nuclear heat shock protein 70 (Hspa1a, Rn00583013_s1) gene. All samples were run in triplicate in 25 µl reaction volume containing 50 ng sample DNA, 2x Probe RT-Mastermix (Qiagen, Valencia, CA, USA), with both probes in each well. The MT_CYB probe was labeled with VIC, and the Hspa1a probe was labeled with FAM. Amplification conditions were: one cycle of 95°C for 15.05 min, and 45 cycles of 95°C for 15 s and 60°C for 1 min and gene expression levels were quantified by the ΔΔCt method.

#### VII.b. RNA quantification

Total RNA was harvested by scraping in ice-cold RNA lysis buffer (200 µL/dish). The SV Total RNA Isolation System kit (Promega, Madison, WI, USA) was used to isolate RNA from neuronal cells following the manufacturer’s instructions. From each sample, 50 pg of total RNA was reverse transcribed and amplified using the Qiagen OneStep Probe RT-PCR Kit (Qiagen). Gene-specific primers targeting rat Drp1 (Dnm1l anti-sense primer/probe, Applied Biosystems: # Rn00586466_m1, VIC) and Beta actin were used (Applied Biosystems: # 4352340E, FAM) with 35 cycles for gene amplification. For the quantification of gene expression levels the ΔΔCt method was used.

### VIII. Statistical Analysis

Statistical analysis was performed with SigmaStat (SPSS, Chicago, IL). Data are presented as means±SEM. Group differences were assessed by one-way ANOVA or two-way repeated-measures ANOVA. The Tukey post hoc test was used for pair wise comparisons. *P*<0.05 was considered statistically significant.

## Results

### Baseline Values

**Figure 1:**
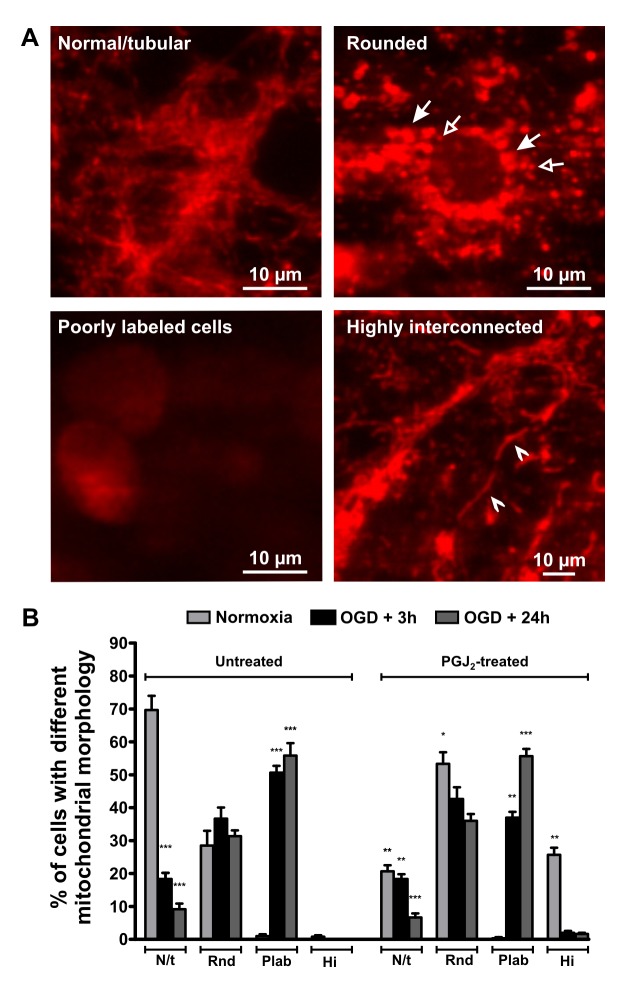
Changes in mitochondrial morphology in neurons following OGD. Representative images of neurons with different mitochondrial morphology with live mitochondrial staining are shown (A). Arrows point to some large, rounded (with filled arrowhead) and small, rounded (with empty arrowhead) mitochondria (top right picture). Poorly labeled cells are shown on the bottom left picture, and arrowheads point to highly interconnected mitochondria on the bottom right picture. The graph (B) shows the percentages of neurons with different forms of mitochondrial morphology in normoxic and OGD conditions with or without PGJ_2_ treatment. An increased percentage of poorly labeled mitochondria containing cells were seen following 3 h OGD with a decreased number of normal, tubular shaped mitochondria. The number of rounded mitochondria containing cells did not change significantly after OGD, having both small and large mitochondria. PGJ_2_ treatment (10 µM for 3 h) increased the ratio of highly interconnected mitochondria in the control cells, but did not affect mitochondrial shape compared to untreated OGD cells. Please note that the y-axis of the graph is the percentage of cells with different mitochondrial morphology and NOT the percentage of mitochondria with different morphology per cell. *p<0.05 **p<0.005 ***p<0.001 vs. untreated control of the same group, n≥3 independent experiments. N/t = Normal/tubular or short/tubular, Rnd = Rounded, Plab = poorly labelled, Hi = Highly interconnected. OGD = oxygen-glucose deprivation, PGJ_2_ = 15-deoxy-D12,14-prostaglandin J_2_.

**Figure 2:**
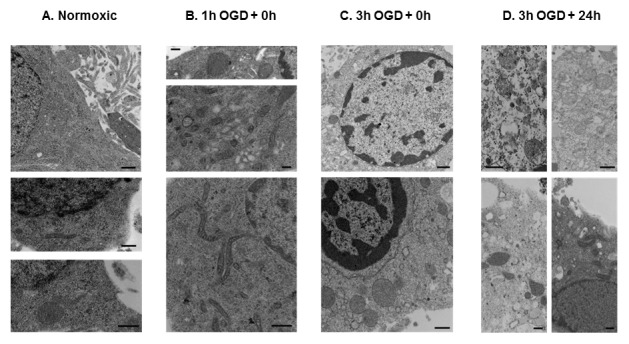
Transmission electron microscopic (TEM) images of mitochondrial changes in neurons after OGD. This figure shows representative TEM images of control and OGD cells. In control cells the mitochondria are mainly small, tubular, with normal density (A, top picture), however, there are some mitochondria that are larger and have normal, or increased intra-mitochondrial density (A, bottom picture). Following 1 h OGD there were several larger and elongated mitochondria in the cortical neurons (B). Several cells also showed the nuclear and cytoplasmic signs of apoptosis (condensed/peripheralized nuclear chromatin and/or cytoplasm, cytoplasmic vacuolization) or necrosis (decreased cytoplasmic density, no detectable plasma membrane) even after 1 h OGD. Following 3 h OGD several mitochondria are large, swollen, show a loss in their internal cristae structure, and their matrix is less dense compared with control (arrowheads). There are, however, several larger, but morphologically intact mitochondria with normal or high density, and some cells have both kinds of mitochondria (Fig. C). Furthermore, most of the cells following 3 h of OGD showed apoptotic or necrotic signs in the TEM pictures. With increasing time after 3 h OGD there were increased numbers of large, swollen mitochondria with rupture/loss of internal cristae (D, arrowheads), whereas some cells still had morphologically intact mitochondria (D, stars). Bars represent 500 nm, stars = mitochondria with intact internal membrane structure, arrowheads = damaged mitochondria, ch = nuclear chromatin, m = mitochondrion, N = nucleus. OGD = oxygen-glucose deprivation.

**Figure 3:**
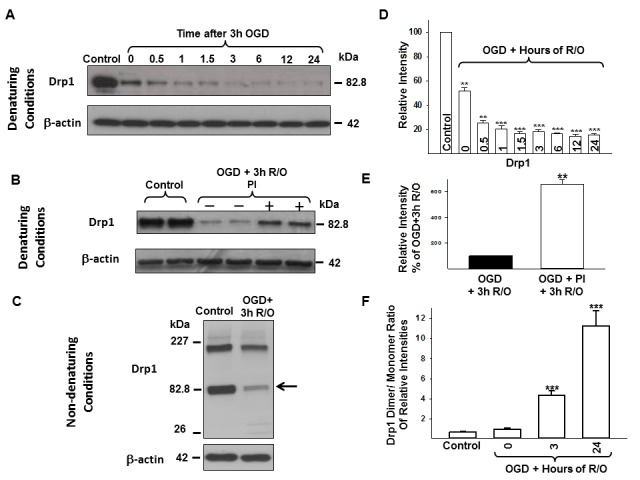
Changes in Drp1 protein expression in neurons following OGD. Representative Drp1 blots are shown with the corresponding β-actin blot using standard western blot protocol and monoclonal Drp1 antibody in control and OGD samples (A) in non-treated and proteinase inhibitor (PI)-treated control and OGD samples (B). Drp1 is highly expressed in neurons under normoxic condition and decreases following 3 h OGD. Proteinase inhibitor treatment results in higher Drp1 expression in OGD cells but is still greatly reduced compared to control. (C) A representative immunoblot with its corresponding β-actin blot using non-denaturing conditions and monoclonal Drp1 antibody. Arrow indicates monomer sized Drp1. Note the two very faint bands of approximately 26 kDa and 32 kDa. Control neurons express high amount of dimer/trimer sized Drp1 that decrease following 3 h OGD. Relative immunoband intensity of Drp1 bands and Drp1 dimer/monomer band ratios are presented as means ± s.e.m. of *n* ≥3 independent experiments (D-E). *p<0.05 **p<0.005 ***p<0.001 vs. control. Drp1 = Dynamin-related protein 1, PI = proteinase inhibitor, OGD = oxygen-glucose deprivation, R/O = reoxygenation.

**Figure 4:**
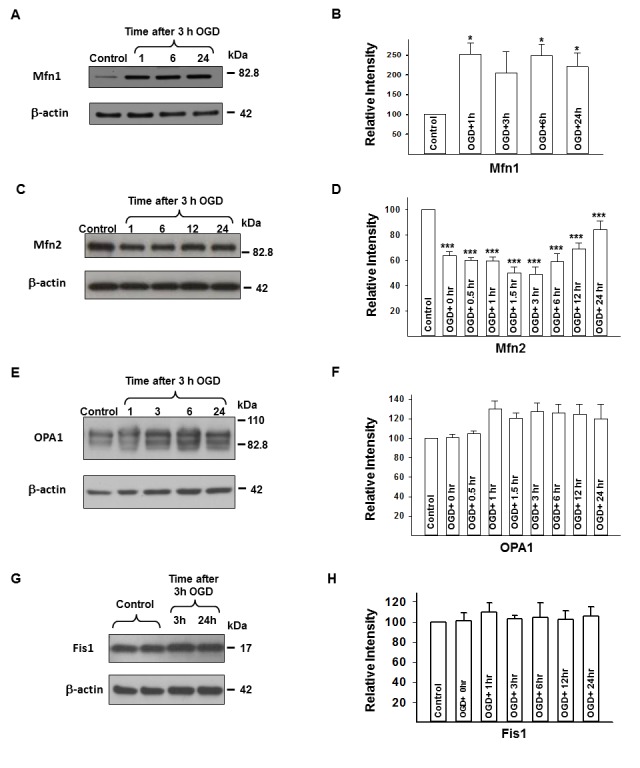
Mfn1, Mfn2, OPA1, and Fis1 protein expression in neurons following OGD. Representative western blots are shown for Mfn1 (A), Mfn2 (C), OPA1 (E), and Fis1 (G) with their corresponding β-actin blots. Panels B, D, F, and H show relative intensity bar graphs for the same proteins as means ± s.e.m. of *n*≥3 independent experiments. Mfn1 increased after OGD, whereas Mfn2 decreased after three, but did not change after one h OGD. OPA1 did not change significantly following OGD. Fis1 protein expression does not change following OGD in neurons. *p<0.05 ***p<0.001 vs. control, *n*≥3 independent experiments. mitofusin1 = Mfn1, mitofusin2 = Mfn2, optic atrophy-1 protein = OPA1, OGD = oxygen-glucose deprivation.

Neurons under normoxic conditions, imaged by live microscopy, primarily exhibited short, tubular mitochondria with a smaller percentage of neurons containing rounded mitochondria, and a few poorly labeled cells (Fig. 1; Fig. 2A). Rounded mitochondria may represent both mitochondrial enlargement or fragmentation depending on their size. Western blots revealed the presence of Drp1 (Fig. 3A), Mfn1 (Fig. 4A), Mfn2 (Fig. 4C), OPA1 (Fig. 4E), and Fis1 (Fig. 4G) in the cell lysates. Drp1 was present as a dimer with molecular weights of approximately 83 kDa and 166 kDa, whereas OPA bands were between 80–100 kDa, as expected; Mfn1, Mfn2, and Fis1 were present only as monomers at appropriate molecular weights.

### One Hour OGD

One hour of OGD followed by 24 h of reoxygenation resulted in a decrease in viability of approximately 20% indicating a moderate degree of cellular stress (data not shown) compared with 3 h OGD where the viability was decreased by more than 50%. Mitochondria became more electron dense with a modest but not significant shift from tubular to large, rounded or elongated configurations (Figure 2B). After 1 h of OGD, the profile of Drp1, Mfn2, OPA1, and Fis1 bands were within normal values after 24 h (data not shown).

### Three Hour OGD

Viability, confocal imaging, transmission electron microscopy (TEM). Three hours of OGD decreased viability of neurons by approximately 60%. Following 3 h of OGD, the percentage of mitochondria exhibiting short, tubular configuration decreased by 80% prior to reoxygenation (p<0.001 in OGD+3 h vs. control), replaced by poorly labeled mitochondria (p<0.001 in OGD+3 h vs. control) as the percentage of rounded mitochondria remained the same (Fig. 1). Small, rounded mitochondria probably result from fragmentation, whereas the larger rounded mitochondria are usually the result of swelling of tubular mitochondria. By 24 h after OGD, very few cells showed short, tubular mitochondria (p<0.001 in OGD+24 h vs. control) with over half of the cell containing primarily poorly labeled (p<0.001 in OGD+24 h vs. control; Fig. 1). Most of the poorly labeled neurons had a physical appearance consistent with cell death (rounded up and contracted), suggesting that these cells are more likely at a later stage of cell death. However, we did not stain them with specific cell death markers. Cells that maintained their processes and thus had normal appearance exhibited primarily rounded or short, tubular mitochondria. These neurons probably formed the surviving cell population, being not at a late stage of cell death.

Using TEM, we detected large, swollen mitochondria as well as normal sized mitochondria but both types had damaged internal membrane structures (rupture or loss of internal cristae) following 3 h of OGD (Fig. 2C/D). In addition, OGD-exposed cells often had numerous large, rounded, but morphologically intact mitochondria. We also detected smaller mitochondria following OGD; however, many of these were outside the cells. These extracellular mitochondria probably were artifacts of the sample preparation: spinning neurons during processing results in loss of axons and dendrites, and severely damaged cells are more likely to lose organelles. Cell nuclei were only found extracellularly in the OGD samples. There were, however, a large number of organelles, mainly mitochondria, outside the cells in the control samples. With increasing reoxygenation time there were a greater number of large, swollen, or normal sized mitochondria with disorganized cristae structure, but many mitochondria still had a morphologically intact shape and higher density (Fig. 2). The discrepancy between these data and our confocal data is probably a result of mitochondrial loss during TEM sample preparation. Mitochondria among the cells on the TEM images were not examined; therefore the changes in the mitochondrial morphology in the whole cell population were calculated from the confocal images. The TEM pictures are, however, essential for determining fine mitochondrial and cell structure.

#### VDAC, complex II, and complex V protein expression, mtDNA

**Figure 5:**
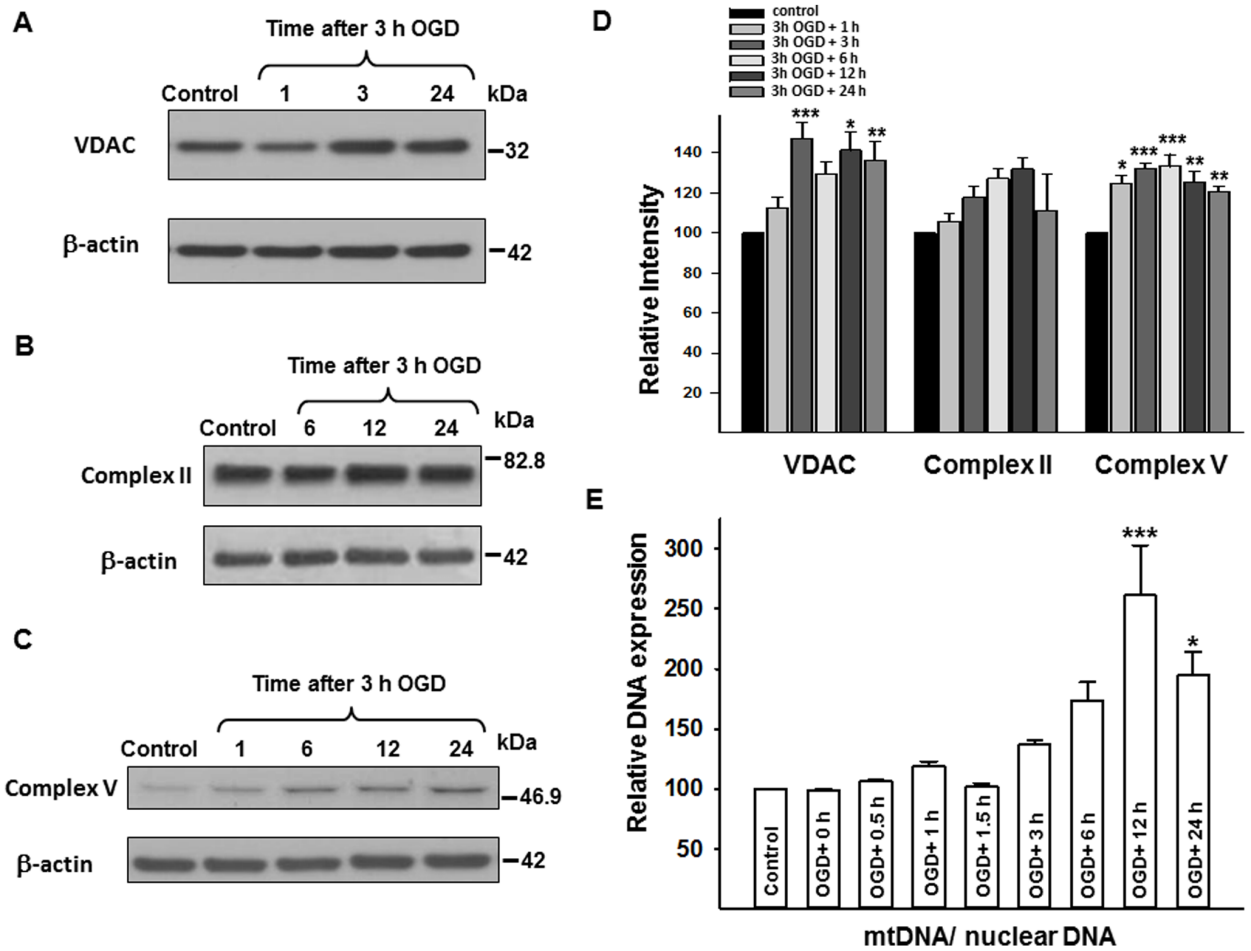
Changes in VDAC, complex II and V proteins, and mtDNA expression following 3 h OGD. Representative western blots for VDAC, Complex II, and Complex V with their corresponding β-actin blots (A, B, C), together with relative intensity values for these proteins (D). VDAC and Complex V proteins showed increased expression, whereas Complex II protein expression did not change significantly following OGD. (E) Shows the changes in mtDNA expression following 3 h of OGD. MtDNA/nuclear DNA ratio significantly increased 12 h following 3 h OGD. *p<0.05 **p<0.005 ***p<0.001 vs. control, n≥3 independent experiments. mtDNA = mitochondrial DNA, OGD = oxygen-glucose deprivation, VDAC = voltage-dependent anion channel.

The VDAC expression was unchanged at 1 h reoxygenation following 3 h OGD but then increased between 3 and 24 hours after reoxygenation (p<0.001 in OGD+3 h; p<0.05 in OGD+12 h; and p<0.005 in OGD+24 h; each vs. control; Fig. 5A/D). The expression of complex II, 70 kDa subunit tended to increase after 3 h OGD with reoxygenation times of 6 and 12 h, but did not reach defined significance (p = 0.059, p = 0.053, respectively; and p = 0.067 for the time series using one-way ANOVA; Fig. 5B/D). Complex V, subunit I expression was significantly increased following OGD (p<0.05 in OGD+1 h; p<0.001 in OGD+3 h and in OGD+6 h; and p<0.005 in OGD+12 h and in OGD+24 h; each vs. control; Fig. 5 C/D). The ratio of mitochondrial DNA (mtDNA) to nuclear DNA expression was significantly increased after OGD with 12 and 24 h reoxygenation (p<0.001 in OGD+12 h vs. control; p<0.05 in OGD+24 h vs. control; Fig. 5E).

#### Expression of fission/fusion proteins

The Drp1 expression decreased by 50% at the end of 3 h of OGD (i.e., 0 h of reoxygenation [p<0.05]) and was further diminished by 3 h reoxygenation when using the monoclonal Drp1 antibody with the standard WB protocol (p<0.005 in OGD+0 and OGD+0.5 h; p<0.001 from 1 to 24 h after OGD; each vs. control; Fig. 3A/D). Since the dramatic disappearance of normal molecular weight by Drp1 during prolonged OGD has not previously been reported, and to reduce the possibility of a technical error, we explored this phenomenon using a proteinase inhibitor and non-denaturing conditions. In addition, we sampled the medium to investigate whether stressed, damaged and/or dying cells release Drp1. Although an inhibitor of proteinase slightly enhanced the band for Drp1 on western blots, the level of Drp1 was still dramatically less following OGD than during normoxic conditions (p<0.005; vs. untreated OGD; Fig. 3B/E). Sampling of the cell culture medium failed to show appreciable Drp1 during or after OGD (data not shown). Nevertheless, using the same antibody with the non-denaturing protocol, two bands were detected in the control samples: a monomer (about 79–84 kDa) and an oligomer sized band (between the 110 kDa and the 227 kDa markers) (Fig. 3C). Similar to the denaturing protocol, we detected a very large decrement in monomer size as well as decreased oligomer band density following OGD using the non-denaturing method (Fig. 3C). The ratio of Drp1 polymer/monomer increased significantly after OGD (p<0.001 at each time point between 3 and 24 h after OGD; each vs. control; Fig. 3F). Furthermore, we detected two very faint bands of approximately 26 kDa and 32 kDa between the 0 and 24 h reoxygenation time points (Fig. 3C). The polyclonal Drp1 antibody showed the same band changes using both standard and non-denaturing protocols as the monoclonal antibody (data not shown). The primary bands representing Drp1 using the monoclonal and polyclonal antibodies had the same molecular weights.

**Figure 6:**
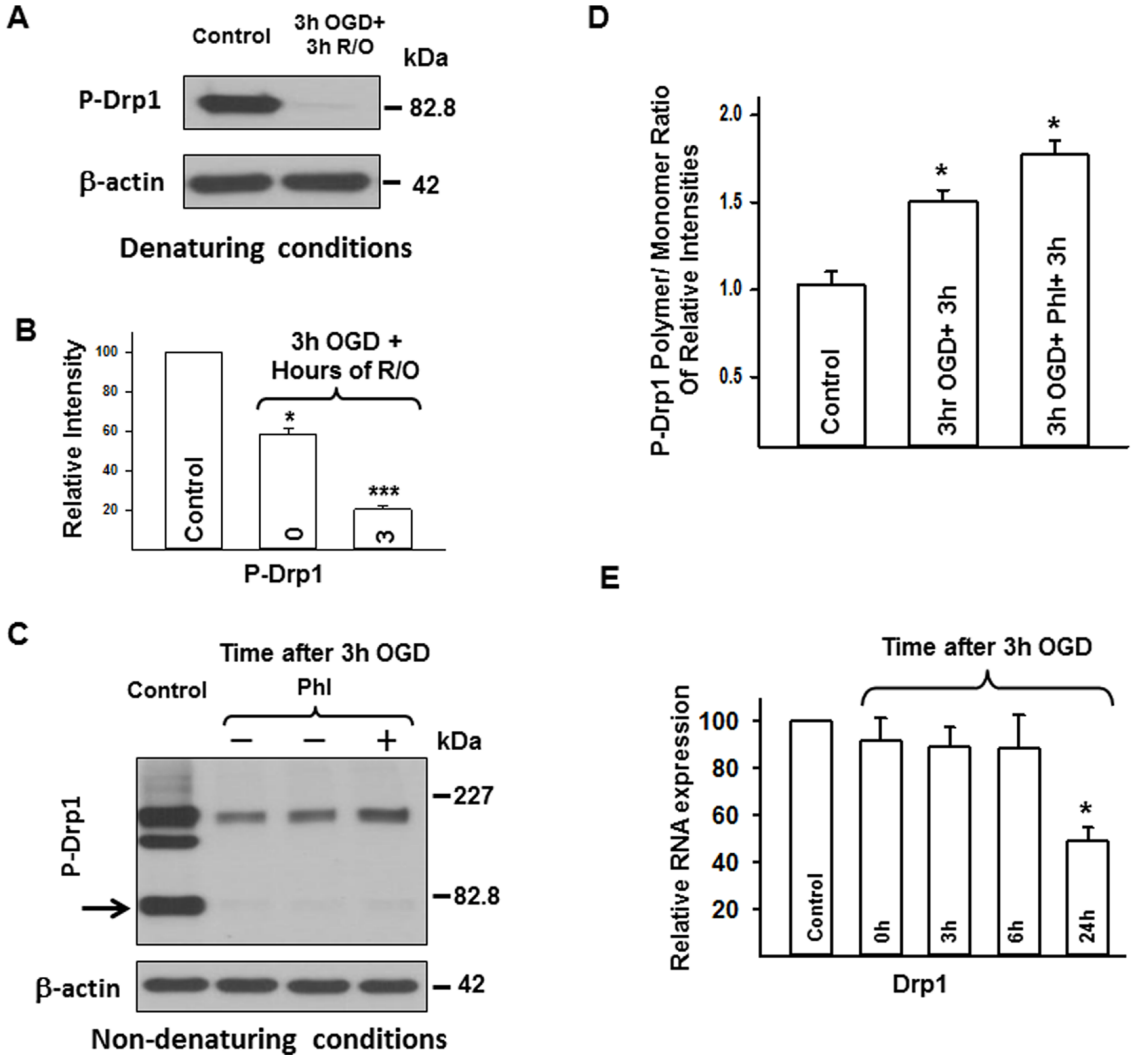
Changes in Ser616 phospho-Drp1 protein, and Drp1 gene expression in neurons following OGD. Representative P-Drp1 immunoblots with the corresponding β-actin blots using standard (A) and non-denaturing (C) western blot protocol in control and OGD samples. P-Drp1 expression is high in control cells but it decreases following 3 h of OGD. (C) The effect of phosphatase inhibitor (PhI) treatment on Ser616 phospho-Drp1 expression. Our PhI-treatment did not increase P-Drp1 expression significantly following 3 h OGD. (B and D) Show the relative immunoband intensity of Ser616 P-Drp1 and the Ser616 P-Drp1 monomer/oligomer ratio; (E) shows the relative Drp1 gene expression, which decreased significantly 24 h after 3 h OGD. *p<0.05 ***p<0.001 vs. control, Drp1 = Dynamin-related protein 1, Fis1 = Fission protein 1, P-Drp1 = Ser616 phospho-Drp1, PhI = phosphatase inhibitor, OGD = oxygen-glucose deprivation, R/O = reoxygenation.

The expression of mRNA for Drp1 did not change during OGD or at 6 h after OGD. However, mRNA for Drp 1 decreased by approximately 50% at 24 h after OGD (Fig. 6E).

In addition to total Drp1, Ser616 phosphorylated Drp1 (P-Drp1) showed decreased expression following 3 h of OGD both under denaturing (p<0.05 at 0 h, p<0.001 3 h after OGD; both vs. control; Fig. 6A/B) and non-denaturing (Fig. 6C) conditions. The ratio of P-Drp1 oligomer/monomer increased significantly after hypoxic stress (p<0.05 at 3 h after OGD; vs. control; Fig. 6D). Addition of the phosphatase inhibitor (5 µl/mL) during OGD slightly increased the expression of the Drp1 oliogomer when compared with OGD alone, however, it was not significant (p = 0.083) and did not preserve the monomer sized band (Fig. 6C). The other mitochondrial fission protein Fis1 expression did not change following OGD (Fig. 4G/H).

The Mfn1 protein expression increased immediately and continued for 24 h after OGD (1, 6, and 24 h of reoxygenation; p<0.05 vs. control; Fig. 4A/B), whereas Mfn2 protein expression decreased following 3 h of OGD (0, 0.5, 1, 3, 6, 12 and 24 h of reoxygenation; each p<0.001 vs. control; Fig. 4C/D). However, 3 h after OGD, Mfn2 protein began to steadily increase towards control values. The expression of OPA1 protein remained unchanged after OGD (p = 0.089 for the time series using one-way ANOVA; Fig. 4E/F).

### PGJ_2_-, and Mdivi- Treatment and OGD

#### Effects on normoxic neurons

**Figure 7:**
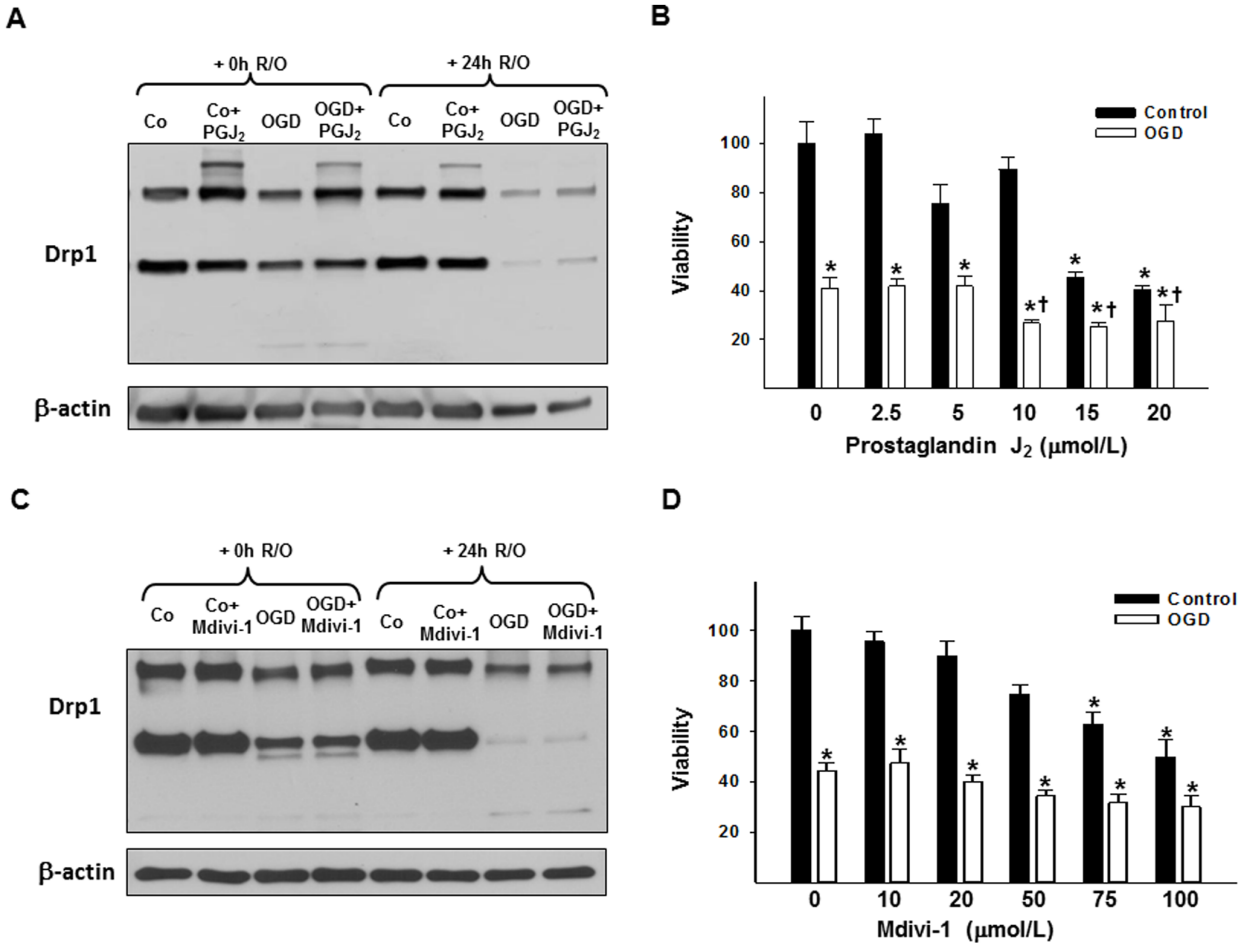
The effect of PGJ2 or Mdivi-1 treatment on cortical neurons under normal and OGD conditions. (A) Shows a representative Drp1 western blot of control, and OGD cells with and without PGJ_2_ treatment. With the non-denaturing western blot method an additional band (above the 227 kDa molecular weight marker) appeared on the western blot due to PGJ_2_ treatment both in control, and in OGD samples, but it was not detectable after 24 h reoxygenation. (B) Shows the changes in viability of the cells due to PGJ_2_ treatment and 3 h OGD. PGJ_2_ treatment decreased cell viability in 15 and 20 µmol/L concentrations and further decreased cell viability after 3 h OGD in 10, 15, and 20 µmol/L concentrations. (C) Shows a representative Drp1 western blot of control, and OGD cells with and without Mdivi-1 treatment. The treatment did not change Drp1 expression in our experiments. (D) Shows the changes in viability of the cells due to Mdivi-1 treatment and 3 h OGD. Mdivi-1 was not protective in 3 h OGD, and at higher doses (75, 100 µmol/L) it decreased cell viability in control neurons. Each panel represents *n*≥3 independent experiments. *p<0.05 vs. control, † p<0.05 vs. untreated OGD, Co = Control, Drp1 = Dynamin-related protein 1, Mdivi-1 = Mitochondrial division inhibitor-1, PGJ_2_ = 15-deoxy-D12,14-prostaglandin J_2_, R/O = reoxygenation, OGD = oxygen-glucose deprivation.

Treatment with PGJ_2_ in control neurons increased oligomerization of Drp1 (Fig. 7A) (Drp1 oligomer/monomer ratio increased, p<0.05 vs. untreated control; data not shown) so that the dimer band was more prominent and an additional prominent band at a higher molecular weight appeared (above the 227 kDa molecular weight marker; Fig. 7/A). Ten µM PGJ_2_ treatment modestly elevated the number of the rounded mitochondria (p<0.05 vs. untreated control) and significantly increased the appearance of higher interconnected mitochondria (p<0.005 vs. untreated control; Fig. 1). Vehicle-treated cells were not different from non-treated cells, therefore it is not shown. The PGJ_2_-treatment also led to cell death at higher concentrations (15 and 20 µmol/L concentrations, both p<0.05; vs. untreated control group), however, it had minimal effects on viability at lower concentrations (Fig. 7B). The Mdivi-1 did not increase expression of Drp 1 (Fig. 7C) but led to neuronal cell death at higher dose concentrations (75–100 µmol/L, both p<0.05; vs. untreated control group; Fig. 7D).

#### Effects during OGD

Viability: PGJ_2_ treatment decreased cell survival beyond OGD alone after 3 h of OGD in 10–15–20 µmol/L concentrations (each p<0.05 vs. untreated OGD group; Fig. 7B), whereas Mdivi-1 treatment did not affect viability significantly following 3 h OGD even at higher concentrations that were toxic under normoxic conditions (Fig. 7D).

Protein expression: The Drp1 expression in neurons exposed to PGJ_2_ was elevated following OGD (p<0.05 vs. untreated OGD) and was similar to levels observed in normoxic neurons treated with PGJ_2_ (data not shown; see Fig. 7A for a representative western blot). An additional band (above the 227 kDa molecular weight marker) was also detected in OGD samples treated with PGJ_2_ at 0 h of reoxygenation, similar to normoxic neurons (Fig. 7A). Furthermore, PGJ_2_ treatment increased the Drp1 oligomer/monomer ratio following OGD as well (p<0.05 vs. untreated OGD; data not shown; see Fig. 7A for a representative western blot). Mdivi-1 treatment did not change Drp1 expression significantly following OGD either (Fig. 7C). Nonetheless, both PGJ2 and mdivi-1 failed to prevent the disappearance of Drp1.

Imaging: Although PGJ_2_ treatment did not cause a major difference in the OGD treated cells concerning viability, there were more rounded and less poorly labeled mitochondria in the PGJ_2_-treated OGD group following 3 h of reoxygenation (both p<0.05 vs. untreated OGD), but not 24 h after reoxygenation (Fig. 1). Morphological changes following Mdivi-1 treatment was not analyzed since we did not find any change in protein expression or cell viability due to the treatment.

## Discussion

The major finding of our study is that mitochondrial biogenesis with maintained fusion is the predominant response in surviving neurons following OGD. Maintained fission was also detected following OGD in our model as several small, rounded (fragmented) mitochondria were seen in the neurons. In addition, this is the first report, which shows high Drp1 polymer expression under normal conditions and Drp1-independent fission associated with mitochondrial fragmentation in neurons following OGD. Our conclusions are based upon these original findings: (1) The percentage of rounded mitochondria stays the same compared with controls following 3 h of OGD despite a dramatic fall in the percentage of short, tubular mitochondria. Large, rounded or tubular mitochondria were typical in the cells with persisting dendrites and/or axons suggesting that certain mitochondrial morphologies, present prior to or occurring following OGD, make the neurons more susceptible or more resilient against OGD-induced stress. (2) The amounts of mitochondrial DNA and electron transport chain proteins increase after OGD in the neurons presumably indicating their attempt to promote mitogenesis, however, mitochondrial turnover was not examined. (3) Maintained mitochondrial fragmentation was detected with confocal imaging. Lastly, (4) the expression of the primary fission protein Drp1 falls dramatically following OGD whereas Mfn1 fusion protein increases. These changes indicate that recovery of neurons following OGD might be restricted by the absence of Drp1 and other fission proteins and is consistent with the retention of large, rounded mitochondria as well as increased levels of mitochondrial DNA and electron transport chain proteins in surviving neurons. It also might indicate that mitochondrial fission occurs in neurons in a Drp-1-independent manner. The lack of protective effect by agents that block Drp1 or inhibit the actions of Drp1 rules out that increased Drp1 expression induces cell death in neurons following OGD. Correcting the imbalance between mitochondrial fission and fusion, however, might represent an important new therapy for limiting damage following ischemic-related events in the brain.

We detected evidence of an intense mitochondrial biogenesis in the neurons following OGD. Thus, the levels of mitochondrial electron transport chain proteins, VDAC, and mtDNA expression increased in the mixed population of surviving and dying cells following OGD, reflecting cell survival efforts involving changes in mitochondrial morphology and function. Additional mitochondrial biogenesis markers, such as peroxisome proliferator-activated receptor-gamma coactivator 1-alpha (PGC-1) or transcription factor A (TFAM), however, were not investigated. We could not exclude the possibility that increased mitochondrial biogenesis markers were not the result of decreased mitophagy and did not investigate electron transport chain complex protein assembly [24]. Even so, our data are most likely explained by the increased need for ATP via induced mitochondrial biogenesis. Mitochondrial fragmentation, possibly involving rapid fission-like events, was observed by confocal imagery, however, both electron microscopic and confocal data revealed the presence of large, morphologically intact mitochondria following OGD. One recent study suggested that induced mitochondrial fission and fusion occur at the same time in the ischemic penumbra in the brain as an effort toward cell survival [7], which supports our observation in cultured neurons. Unfortunately, methodological limitations did not allow us to differentiate between anoxia-resistant/−susceptible mitochondria during and following OGD. The rapid fall in Drp1 during, and especially following OGD indicate that the Drp1-dependent fission is minimal during the post-OGD period.

The Drp1 protein expression never recovered from its dramatic fall even in surviving neurons following OGD in our study. We examined this phenomenon further to explore the mechanisms of Drp1 degradation and to minimize the possibility of an artifact related to our methods. The addition of a proteinase inhibitor partially preserved Drp1 expression indicating protein degradation during and after 3 h OGD in primary neurons. Possible degradation of Drp1 was further supported by the lower molecular weight bands detected on western blots. However, the density of the lower molecular weight bands did not approximate the original Drp1 bands and no detectable Drp1 was present in the medium. Consequently, we conclude that massive, rapid Drp1 degradation occurs along with other structural changes in the non-degraded Drp1, which does not allow protein detection with our antibodies. Furthermore, the surviving neurons apparently do not support Drp1 expression, since mRNA expression levels also fall by 24 h post OGD. Structural change during OGD, however, might be involved in cell death induction. Increased oligomer/monomer ratio following OGD was also seen, but interpretation is difficult because of the greatly reduced amount of total Drp1. According to previous studies, increased or unchanged Drp1 expression, Drp1 translocation to the mitochondria, and/or increased oligomer/monomer ratio is accompanied by cell death [15], [19], [25]–[27]. Conversely, previous studies showed that cell death induction and mitochondrial fragmentation occur without Drp1 [28], [29], and mitochondrial fission itself can be Drp1-independent [30]. In addition, reduced but not increased Drp1 expression is known to induce cell death in neurons showing the underlying need for high Drp1 expression to maintain viability [2].

We investigated Ser616 phosphorylation of Drp1 following 3 h OGD because growing evidence suggests that phosphorylation is an important determinant of mitochondrial fission activity [3], [6]. Phosphorylation on Ser616 or Ser579 sites increases Drp1 activity [25], [31], whereas Ser637/Ser656 phosphorylation reduces mitochondrial fission by inhibition of GTPase activity [3], [6], [7]. These modifications may be of low importance because of their reliance on studies showing no change in mitochondrial dynamics with altered Drp1 phosphorylation state [5], [32]. In our experiments, however, it is unlikely that the significantly decreased Drp1 and P-Drp1 expression were compensated by increased activity through other post translational modifications since total Drp1 levels fall tremendously following OGD. Altogether, Drp1 does not appear to be a key molecule in mitochondrial fission or cell death following OGD in neurons.

To investigate the mitochondrial target of Drp1 we measured Fis1 expression, which did not change following OGD. Therefore, in our OGD model, Fis1 is either not a key molecule in mitochondrial fission or its unchanged expression is able to maintain high fission activity. Nevertheless, the importance of Fis1 both in mitochondrial fission and in cell death induction is controversial [33]–[35]. Furthermore, there are several newly identified proteins in mitochondrial fission that are thought to be involved in Drp1-mediated fission rather than Fis1 [8], [33] and some studies postulate the existence of unidentified mammalian proteins that regulate mitochondrial dynamics [36], [37].

To further investigate the role of Drp1 in neurons following OGD, we used two different Drp1-blockers, PGJ_2_ and Mdivi-1, but neither of these drugs improved cell viability in our experimental setup. The PGJ_2_, however, significantly increased Drp1 protein expression and polymerization, and also induced mitochondrial fusion, as shown on other cell types [29]. In addition to its peroxisome proliferator-activated receptor gamma (PPARγ) activator effect, PGJ_2_ also blocks Drp1 activity in a higher dose range in a PPARγ-independent manner [29] as do other PPARγ-idependent effects that are mediated through other pathways, such as Janus kinase (JAK) or STAT [38]. Data on the drug’s effect is, nevertheless, controversial. The PGJ_2_ can induce or inhibit cell proliferation/angiogenesis [39], [40], can decrease [38], [41], [42] or activate [41] inflammatory response, and can increase cell survival following some injuries [21], [43] but not others [20], [44]. The Mdivi-1, which is known to inhibit Drp1effectiveness [45], [46], did not change protein expression or cell viability in our experiments. In contrast to our results, a few recent studies showed increased cell survival in neurons and endothelial cells following OGD due to Drp1 effects [15], [19]. Differences in the experimental setup and cell viability measurement method may be the reason for the contradictory data in neurons; whereas, in endothelial cells, increased Drp1 expression following stress might represent a cell type-specific effect. Our data from the putative blockers lend further support to the view that Drp1 is not a key regulator in cell death induction following OGD in neurons.

To investigate mitochondrial dynamics from the fusion side we studied Mfn1, Mfn2, and OPA1 expression. In stark contrast to Drp1, Mfn1 showed an increase after OGD, whereas Mfn2 decreased by approximately 50% after OGD, but was restored to near control values by 24 h. OPA1 expression remained unchanged following OGD. Mitofusins and OPA1 are thought to be involved in mitochondrial external and internal mitochondrial membrane fusion, respectively [47], [48]. In contrast with Mfn2, which is also an important signaling molecule, Mfn1 and OPA1 play a more direct role in mitochondrial docking and fusion [48]–[50]. The exact role of the altered fusion protein expression in neuronal cell death is unclear, and there are conflicting data, especially whether Mfn2 has effects on cell survival. On one hand, increased Mfn2 expression is associated with apoptosis [51], [52]. On the other hand, reduced Mfn2 expression can aggravate cell damage [49], [53], [54], but its increased expression can be protective in other models [18], [55]. The OPA1 regulates mitochondrial cristae remodeling independently of its effect on mitochondrial fusion [56], facilitating and accelerating cytochrome c release during apoptosis [57]. OPA1 is also known to increase in the ischemic core following experimental focal brain ischemia [7]. The lower Mfn2 expression and maintained OPA1 expression seem to play a role in the neuronal cell death process in our model. The increased Mfn1 expression shown in our study may reflect ongoing mitochondrial fusion, which might represent an attempt by mitochondria to maintain energy production following OGD.

We also examined the effect of short-term (1 h) OGD in primary cortical neurons in order to observe the effect of milder stress on mitochondrial dynamics. Surprisingly, 1 h OGD did not change Drp1, Fis1, Mfn2, or OPA1 expression in our model. In contrast, mitochondria often appeared to be larger and denser compared with controls. This finding indicates the involvement of other mitochondrial fission-fusion proteins or post-translational modifications that regulate mitochondrial dynamics.

In conclusion, our findings indicate that both fission and fusion driven mechanisms are predominant responses to prolonged OGD in primary cultures of rat neurons. Thus, Drp1 does not appear to be a major regulator of altered mitochondrial dynamics following long term OGD in primary cortical neurons. However, Mfn1 fusion protein, which increased following OGD, may be a regulator of the attempt for cell survival. An imbalance favoring fusion over fission appears to be beneficial in response to OGD in neurons. Further studies are needed to determine whether increased mitochondrial fusion can increase neuronal survival.
